# Culture yield of repeat percutaneous image-guided biopsy after a negative initial biopsy in suspected spondylodiscitis: a systematic review

**DOI:** 10.1007/s00256-018-3006-5

**Published:** 2018-06-18

**Authors:** Ömer Kasalak, Hugo J. A. Adams, Paul C. Jutte, Jelle Overbosch, Rudi A. J. O. Dierckx, Marjan Wouthuyzen-Bakker, Thomas C. Kwee

**Affiliations:** 1 Department of Radiology, Nuclear Medicine and Molecular Imaging, University Medical Center Groningen, University of Groningen, Groningen, The Netherlands; 2 Department of Radiology and Nuclear Imaging, Deventer Hospital, Deventer, The Netherlands; 3 Department of Orthopedics, University Medical Center Groningen, University of Groningen, Groningen, The Netherlands; 4 Department of Medical Microbiology and Infection Prevention, University Medical Center Groningen, University of Groningen, Groningen, The Netherlands

**Keywords:** Biopsy, CT, Culture yield, Spine infection, Spondylodiscitis

## Abstract

**Objective:**

To systematically review the published data on the culture yield of a repeat (second) percutaneous image-guided biopsy after negative initial biopsy in suspected spondylodiscitis.

**Materials and methods:**

A systematic search was performed of the PubMed/Medline and Embase databases. The methodological quality of the studies included was assessed. The proportions of positive cultures among all initial biopsies and second biopsies (after a negative initial biopsy) were calculated for each study and assessed for heterogeneity (defined as I^2^ > 50%).

**Results:**

Eight studies, comprising a total of 107 patients who underwent a second percutaneous image-guided biopsy after a culture-negative initial biopsy in suspected spondylodiscitis, were included. All eight studies were at risk of bias and were concerning with regard to applicability, particularly patient selection, flow of patients through the study, and timing of the biopsy. The proportions of positive cultures among all initial biopsies ranged from 10.3 to 52.5%, and were subject to heterogeneity (I^2^ = 73.7%). The proportions of positive cultures among all second biopsies after negative initial biopsy ranged from 0 to 60.0%, and were not subject to heterogeneity (I^2^ = 38.7%).

**Conclusion:**

Although a second percutaneous image-guided biopsy may have some value in patients with suspected spondylodiscitis, its exact value remains unclear, given the available poor-quality evidence. Future well-designed studies are needed to determine the role of a second percutaneous image-guided biopsy in this setting. Such studies should clearly describe the spectrum of patients that was selected for a second percutaneous image-guided biopsy, the method of biopsy, and differences compared with the first biopsy, if any.

## Introduction

Spondylodiscitis refers to infection of the vertebrae and intervertebral disc [1]. The incidence of spondylodiscitis is approximately 2.4 cases per 100,000 population [2, 3], and is on a steady rise owing to an aging population with inherent co-morbidities, and improved case ascertainment, particularly related to the widespread use of magnetic resonance imaging (MRI) [1]. Because of its nonspecific presentation, a delay of 6–8 weeks between the onset of symptoms and diagnosis is not unusual [1, 2]. However, because spondylodiscitis can be complicated by abscess formation, orthopedic complications (vertebral collapse and hyperkyphosis), neurological complications (motor weakness or paralysis), and even death (in approximately 6%) [1, 2], timely diagnosis and treatment initiation are essential. Current Infectious Diseases Society of America (IDSA) guidelines recommend performing spine MRI and obtaining blood cultures in all patients with suspected spondylodiscitis [4–6]. The same guidelines also recommend an image-guided biopsy when a microbiological diagnosis has not been established by blood cultures or serological tests [4, 5]. A recent meta-analysis showed the culture yield of the initial image-guided biopsy in spondylodiscitis to be approximately 48% [7]. However, when both blood and biopsy cultures remain negative, it is unclear whether empirical antibiotic therapy should be started, if a repeat (second) image-guided biopsy should be performed, or if more invasive procedures such as percutaneous endoscopic discectomy and drainage (PEDD) or open excisional biopsy should be considered [7]. The advantage of a second image-guided biopsy is that it is less invasive than PEDD or an open excisional biopsy. However, because of the limited number of studies on this topic with relatively small sample sizes and heterogeneous methodology, the culture yield of a second image-guided biopsy is still unclear. Theoretically, if the patient spectrum and technical factors related to the biopsy are the same for the first and second attempts, the culture yields will be the same. However, this may not be the case in clinical practice. Information on the culture yield of a second image-guided biopsy, as performed in clinical practice, is crucial for evidence-based clinical decision-making. Therefore, the aim of this study was to systematically review published data on the culture yield of a second percutaneous image-guided biopsy after a negative initial biopsy in patients with suspected spondylodiscitis.

## Materials and methods

### Search strategy

The PubMed/Medline and Embase databases were systematically searched for articles on the culture yield of a second percutaneous image-guided biopsy after a negative initial biopsy in suspected spondylodiscitis. The search comprised a combination of the search terms “spondylodiscitis OR spondylodiskitis OR discitis OR diskitis OR spondylitis OR spinal osteomyelitis OR vertebral osteomyelitis” AND “biopsy OR biopsies OR aspiration OR aspirations OR sample OR samples OR sampling OR samplings” AND “computed tomography OR computerized tomography OR CT OR CT-guided OR fluoroscopic OR percutaneous.” No date restriction was applied. The search was updated to 18 November 2017. References of articles that remained after the selection process were screened for potentially suitable additional articles.

### Study selection

Studies investigating the culture yield of a second percutaneous image-guided biopsy after a negative initial biopsy in suspected spondylodiscitis were eligible for inclusion. No language restriction was applied. Conference abstracts, case reports or series, editorials or letters, review articles, and meta-analyses were excluded. Articles that only reported the culture yield of the initial percutaneous, image-guided biopsy and that not did report or allow for the extraction of the culture yield of the second biopsy after a negative initial biopsy, were excluded. Articles that only included patients who underwent aspiration of postoperative paraspinal fluid collections or PEDD were excluded. When the same patient data were presented in more than one article, the article with the largest number of patients was selected. Three researchers (Ö.K, H.J.A.A., and T.C.K.) reviewed the titles and abstracts of the retrieved articles in consensus, applying the previously mentioned inclusion and exclusion criteria. Clearly ineligible articles were excluded at this stage. Subsequently, the same three researchers jointly reviewed the full text version of the remaining articles to determine their eligibility for inclusion.

### Study quality

Methodological quality of the studies included was assessed using the Quality Assessment of Studies of Diagnostic Accuracy Included in Systematic Reviews (QUADAS)-2 tool [8]. The QUADAS-2 tool comprises four domains: patient selection, index test, reference standard, and flow and timing [8]. Each domain is assessed in terms of risk of bias, and the first three domains are also assessed in terms of concerns regarding applicability [8]. In the present study, image-guided biopsy and microbiological culture can be considered as both index test and reference test. Therefore, index test and reference standard were combined into one domain called “biopsy” for both risk of bias and applicability assessment. Risk of bias and concerns about applicability for each domain were judged to be “high,” “unclear,” or “low.” If a study is judged as “low” on all domains relating to bias or applicability, then it is appropriate to have an overall judgment of “low risk of bias” or “low concern regarding applicability” for that study [8]. If a study is judged to be “high” or “unclear” in one or more domains, then it may be judged to be “at risk of bias” or as having “concerns regarding applicability” [8].

### Statistical analysis

The proportions of positive cultures (i.e., cultures with isolated bacteria in the biopsy) among all initial biopsies and second biopsies (after a negative initial biopsy) were calculated for each individual study. Heterogeneity in positive culture yields across individual studies was assessed using the I^2^ statistic, with heterogeneity defined as I^2^ > 50% [9]. Statistical analyses were performed using Comprehensive Meta-Analysis Version 3 software (Biostat, Englewood, IL, USA).

## Results

### Literature search

The systematic search yielded 1,300 articles from PubMed/Medline and 920 articles from Embase. After reviewing titles and abstracts, 78 PubMed/Medline indexed articles and 62 Embase indexed articles remained. After discarding duplicates, 93 potentially eligible articles remained, and these were retrieved in full-text format. After reviewing the full-text article, 85 articles were excluded because they only reported the culture yield of the initial percutaneous image-guided biopsy and did not report or allow for the extraction of the culture yield of the repeat biopsy after a negative initial biopsy. Finally, eight studies remained [10–17], comprising a total of 107 patients who underwent a second percutaneous image-guided biopsy after a negative initial biopsy in suspected spondylodiscitis. The characteristics of these studies are displayed in Tables 1 and 2.

**Table 1:** Characteristics of the studies and patients included

Study	Country	Data acquisition	Number of patients^a^	Age in years (range)^a^	Gender (male/female)^a^	Patient spectrum^a^	CRP levels in mg/L (range)^a^	Leukocyte count ×10^9^ (range)^a^
Ahuja and Sharma [10]	UK	Retrospective	45	62.3^b^ (28–87)	26/19	Patients with suspected spinal infection based on clinical and MRI findings Postoperative spinal infections were excluded	58^c^ (30–90^d^)	8.46^b^ (2.4–9)
Terreaux et al. [11]	France	Retrospective	63	68.2^b^ (NR)	34/29	Patients with spontaneous spondylodiscitis and negative blood cultures Patients with postoperative spondylodiscitis, positive blood cultures before the development of spontaneous spondylodiscitis, spontaneous discitis with negative blood cultures investigated by surgical biopsy, tumors, and crystal deposition disease were excluded	NR	NR
Gras et al. [12]	France	Retrospective	136	58^c^ (47–69^d^)	89/47	Hospitalized patients ≥18 years with spondylodiscitis, pre-biopsy negative blood culture(s), CT-guided biopsy by an interventional radiologist and one or more post-biopsy blood cultures (0–4 h) Patients with continuous osteomyelitis due to decubitus ulcers, a vertebral device, brucellosis, and tuberculous spondylodiscitis were excluded Patients receiving antibiotics within 2 weeks preceding the biopsy were also excluded	NR	NR
Kim et al. [13]	South Korea	Retrospective	140	65.1^b^ (16–89)	70/70	Patients in whom fluoroscopy-guided biopsy was performed to confirm or rule out the clinical or radiological possibility of spondylodiscitis Patients who underwent biopsy for suspected primary bone tumor or metastases were excluded	NR	NR
Gasbarrini et al. [14]	Italy	Prospective	69	60^b^ (5–85)	37/32	Patients in whom CT-guided biopsy was performed in the case of infection indicated on MRI, elevated inflammation markers (ESR, CRP), with a thoracic, lumbar or sacral lesion (cervical lesions were excluded), the absence of bacteriological isolation elsewhere, the absence of indication for emergency surgery, and in whom no antibiotic therapy was initiated or who were outside the therapeutic window of a previously taken antibiotic	NR	NR
Lora-Tamayo et al. [15]	Spain	Retrospective	72	66 (NR)	43/29	Patients with pyogenic spondylodiscitis Postsurgical cases of pyogenic osteomyelitis, cases of facet joint infection with no involvement of the intervertebral disc or vertebral bodies, and cases due to *Mycobacterium tuberculosis*, *Brucella* species, and fungus were excluded	NR	NR
De Lucas et al. [16]	Spain	Retrospective	40	58^b^ (1–88)	24/16	Patients with confirmed spondylodiscitis, based on imaging findings, positive cultures from CT-guided or surgical biopsy, or blood samples and satisfactory evolution after antibiotic treatment	NR	NR
Friedman et al. [17]	USA	Retrospective	48	68.2^b^ (NR)	26/22	Adult patients with spontaneous infectious spondylodiscitis who were treated by a single surgeon over a 5-year period and patients with postoperative discitis over the same time period	NR	NR

*CRP* C-reactive protein, *NR* not reported

^a^(Based on the) total number of patients that was included in this study

^b^Mean

^c^Median

^d^Interquartile range

**Table 2:** Magnetic resonance imaging and biopsy methods

Study	Availability of MRI before tissue sampling	MRI criteria for spondylodiscitis	MRI readers	Time between MRI and biopsy	Time between the initial and second biopsies	Type of image guidance for biopsy	Gauge, number of samples	Tissue targeted	Second biopsy of the same area as first biopsy	Physician(s) who performed biopsy
Ahuja and Sharma [10]	Yes	NR	NR	NR	NR	CT	NR, NR	NR	NR	Radiologist
Terreaux et al. [11]	Yes (in 60/63 [95%])	NR	NR	NR	14.4^a^ ± 7.9	CT or fluoroscopic	11 to 14, NR	Disc	NR	NR
Gras et al. [12]	NR	NR	NR	NR	NR	CT	NR, 2.5^a^	Vertebral corpus	NR	Interventional radiologist
Kim et al. [13]	NR	NR	NR	NR	NR	Fluoroscopic	15, >2	Vertebral corpus/disc/paravertebral abscess	22/26 same area 4/26 different area	NR
Gasbarrini et al. [14]	Yes	Hypointense on T1, hyperintense on T2, morphologically consistent with infection	NR	NR	NR	CT	8 or 11, NR	Bone/disc	NR	Both interventional radiologist and surgeon, whenever possible
Lora-Tamayo et al. [15]	NR	NR	NR	NR	NR	CT	13.55 to 22^b^, NR	Vertebral corpus/disc/abscess/paraspinal phlegmon	NR	Musculoskeletal radiologists
De Lucas et al. [16]	Yes (in 32/40 [80%])	NR	Radiologist	NR	NR	CT	20–22, NA^c^	Vertebral corpus/disc/paravertebral soft tissue/abscess	NR	NR
Friedman et al. [17]	NR	NR	NR	NR	NR	NR	NR	Disc	NR	NR

*CT* computed tomography, *MRI* magnetic resonance imaging, *NR* not reported, *NA* not applicable

^a^Mean

^b^Both biopsies and aspirations were performed

^c^Aspiration

### Methodological quality assessment

Results of the quality assessment using the QUADAS-2 tool are displayed in Table 3. Overall, all studies were at risk of bias and all studies had concerns regarding applicability. There was a high risk of bias in the domain patient selection in 7 studies [10–16], because only a minority of patients with negative initial biopsy cultures underwent a second biopsy and it was unclear why these patients were selected for repeat biopsy. There was an unclear risk of bias and applicability concern in the domain of biopsy in 7 studies, because they did not describe sufficient details on how percutaneous image-guided biopsy was performed in terms of image guidance, needle size, and number of biopsy samples acquired [10–12, 14–17]. There was unclear risk of bias in the domain of flow and timing in all 8 studies [10–17], because of the time frame between MRI, the first biopsies and second biopsies were not described. In addition, in 7 studies it was unclear if patients received antibiotic treatment between the first and second biopsies [10–15, 17]. There was an unclear applicability concern in the domain of patient selection in all 8 studies [10–17], because it was unclear if patients with a previous history of spondylodiscitis were included, which patients were selected for second biopsy, and which of these patients had already been treated with antibiotics. In addition, in 4 studies it was unclear if MRI had been performed at all [12, 13, 15, 17], in 4 other studies no (clear) MRI criteria for spondylodiscitis were reported [10, 11, 14, 16], and in 2 studies it was unclear whether patients with positive blood cultures before biopsy were excluded [10, 16].

**Table 3:** Quality assessment of the studies included using the QUADAS-2 tool [8]

Study	Risk of bias			Applicability concerns	
	Patient selection	Biopsy	Flow and timing	Patient selection	Biopsy
Ahuja and Sharma [10]	High	Low	Unclear	Unclear	Low
Terreaux et al. [11]	High	Low	Unclear	Unclear	Low
Gras et al. [12]	High	Low	Unclear	Unclear	Low
Kim et al. [13]	High	Low	Unclear	Unclear	Low
Gasbarrini et al. [14]	High	Low	Unclear	Unclear	Low
Lora-Tamayo et al. [15]	High	Low	Unclear	Unclear	Low
De Lucas et al. [16]	High	Low	Unclear	Unclear	Low
Friedman et al. [17]	Low	Unclear	Unclear	Unclear	Unclear

The following signaling questions were used to assess the risk of bias and applicability concerns (which were then scored as high risk, low risk, or unclear):

Risk of bias:

1. Patient selection. Did most patients with negative initial biopsy cultures undergo a repeat biopsy? Was it reported why patients were selected for repeat biopsy?

2. Biopsy. Could the conduct or interpretation of biopsy have introduced bias?

3. Flow and timing. Was MRI performed within 2 months before tissue biopsy? Was the repeat biopsy performed within 1 month of the initial biopsy and was no therapy administered between the initial and repeat biopsies?

Applicability concerns:

4. Patient selection. Were patients with a previous history of spondylodiscitis excluded? Were patients with positive blood cultures before biopsy excluded? Was MRI performed before biopsy and were the criteria for positivity reported? Which patients underwent a repeat biopsy after a negative initial biopsy?

5. Biopsy. Was fluoroscopic or CT guidance used? What needle size was used? How many biopsy samples were acquired?

### Culture yield of the repeat biopsy

**Fig. 1:**
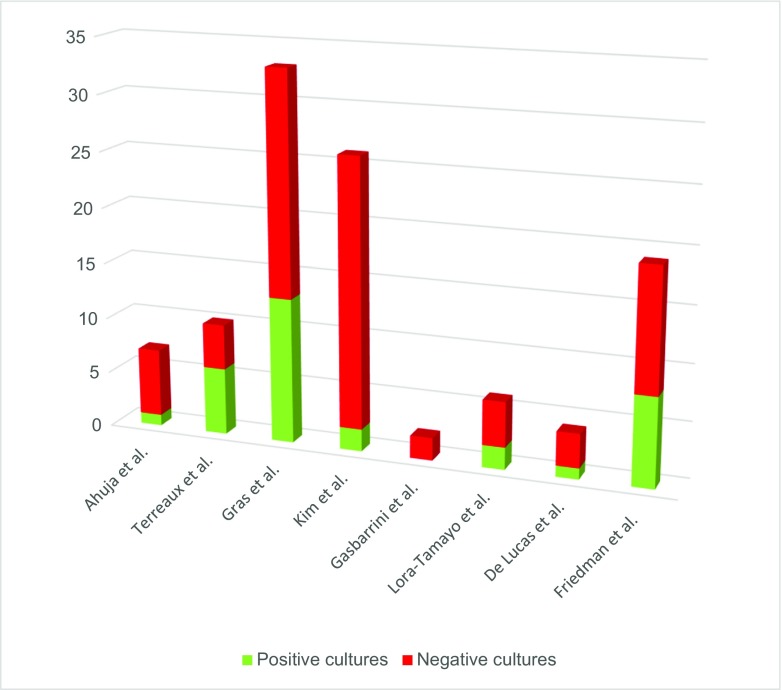
Number of positive and negative cultures on the repeat biopsy (after a negative initial biopsy) in suspected spondylodiscitis, for each of the eight studies included

Culture yields of initial and second biopsies for each individual study are displayed in Table 4. The proportions of positive cultures among all initial biopsies (*n* = 507) ranged from 10.3 to 52.5% (with I^2^ = 73.7%). The proportions of positive cultures among all second biopsies (*n* = 107) ranged from 0 to 60.0% (with I^2^ = 38.7%; Fig. 1).

**Table 4:** Results of included studies

Study	Number of culture-positive initial biopsies	Cultured micro-organisms of the initial biopsy	Number of culture-positive second biopsies after a negative initial biopsy^c^	Cultured micro-organisms on repeat biopsy^c^
Ahuja and Sharma [10]	19/45 (42.2%)	*Escherichia coli* (*n* = 2) *Propronibacterium acnes* (*n* = 2) *Staphylococcus aureus* (*n* = 2) *Candida albicans* (*n* = 1) Coagulase-negative staphylococci (*n* = 1) *Enterococcus faecium* (*n* = 1) Group B hemolytic streptococci (*n* = 1) *Klebsiella oxytoca* (*n* = 1) Methicillin-resistant* Staphylococcus aureus* (*n* = 1) *Mycobacterium tuberculosis* = (*n* = 1) *Pseudomonas aeruginosa* = (*n* = 1) *Staphylococcus capitis* = (*n* = 1) *Streptococcus sanguinis* = (*n* = 1) *Staphylococcus aureus* and *Propionibacterium acnes* (*n* = 1) Coagulase-negative staphylococci, *Propionibacterium acnes*, and *Streptococcus mutans* (*n* = 1) Nonhemolytic streptococci and* Propionibacterium* spp. (*n* = 1)	1/7 (14.3%)	*Staphylococcus epidermidis* and *Propionibacterium acnes* (*n* = 1)
Terreaux et al. [11]	33/63 (52.4%)	Methicillin-susceptible* Staphylococcus aureus* (*n* = 9) *Staphylococcus epidermidis* (*n* = 8) *Mycobacterium tuberculosis* (*n* = 3) *Streptococcus constellatus* (*n* = 3) *Propionibacterium acnes* = (*n* = 2) Methicillin-resistant* Staphylococcus aureus* (*n* = 1) *Enterococcus hirae* (*n* = 1) *Klebsiella pneumoniae* (*n* = 1) *Staphylococcus caprae* (*n* = 1) *Streptococcus mutans* (*n* = 1) *Streptococcus dysgalactiae* (*n* = 1) *Streptococcus milleri* type 2 (*n* = 1) *Escherichia coli* (*n* = 1)	6/10 (60.0%)	*Streptococcus* (*n* = 3) *Prevotella denticola* (*n* = 1) *Pseudomonas aeruginosa* (*n* = 1) *Mycobacterium tuberculosis* (*n* = 1)
Gras et al. [12]	59/136 (43.4%)	NR	13/33 (39.4%)	NR
Kim et al. [13]	51/170 (30.0%)	*Mycobacterium tuberculosis* (*n* = 26) *Staphylococcus aureus* (*n* = 13) *Streptococcus agalactiae* (*n* = 4) *Streptococcus viridans* (*n* = 2) Coagulase-negative* Staphylococcus* organisms (*n* = 1) *Escherichia coli* (*n* = 1) *Enterococcus faecalis* (*n* = 1) *Enterobacter cloacae* (*n* = 1) *Staphylococcus epididymis* (*n* = 1) *Klebsiella* (*n* = 1)	2/26 (7.7%)	*Mycobacterium tuberculosis* (*n* = 1) *Staphylococcus aureus* (*n* = 1)
Gasbarrini et al. [14]	11/24 (45.8%)^a^	*Mycobacterium tuberculosis* (*n* = 3) *Staphylococcus hominis* (*n* = 2) Methicillin-resistant* Staphylococcus aureus* (*n* = 1) Methicillin-susceptible* Staphylococcus aureus* (*n* = 1) *Streptococcus* spp. (*n* = 1) *Streptococcus constellatus* (*n* = 1) *Pseudomonas aeruginosa* (*n* = 1) *Escherichia coli* (*n* = 1)	0/2 (0.0%)	NA
Lora-Tamayo et al. [15]	3/29 (10.3%)	NR	2/6 (33.3%)	NR
De Lucas et al. [16]	NR^b^	NR	1/4 (25.0%)^d^	*Mycobacterium tuberculosis* (*n* = 1)
Friedman et al. [17]	21/40 (52.5%)	NR	8/19 (42.1%)	NR

*NR* not reported, *NA* not applicable

^a^Including one case that was culture-negative, but in whom *Mycobacterium tuberculosis* was molecularly detected by polymerase chain reaction

^b^Initial and second CT-guided biopsy results could not be separated with certainty

^c^No diagnoses other than spondylodiscitis (if present) were reported after initial or second biopsy

^d^Antibiotics were given after the first negative biopsy in 3 cases, and no antibiotics were given to the other case, which turned out to be culture-positive

## Discussion

This systematic review included 8 studies, comprising a total sample size of 107 patients who underwent a second image-guided biopsy after a culture-negative initial biopsy in suspected spondylodiscitis. The positive culture yield of a second CT-guided biopsy ranged between approximately 10 and 50% of the studies included. However, these percentages should be interpreted cautiously because the overall quality of the studies included was poor to moderate, with several important methodological concerns. First, in 7 studies, only a minority of patients with a negative initial biopsy cultures underwent a second biopsy [10–16]. As 6 of these 7 studies were performed retrospectively [10–13, 15, 16], the decision to rebiopsy was most likely based on clinical and radiological grounds, which may include insufficient treatment response and disease extent on MRI (e.g., the presence of large paravertebral phlegmon and/or abscess). These factors might overestimate the culture yield if extrapolated to all patients with a negative initial biopsy culture. Although the culture-positive rebiopsy yields do reflect clinical practice, the exact reasons why the patients in these studies were rebiopsied, remain unclear. Furthermore, from the 8 studies included, it was unclear how many of the patients with culture-negative initial biopsies eventually had positive blood cultures, how many underwent PEDD or open biopsy, and how many received empirical antibiotic therapy without any further diagnostic interventions [10–17]. Second, there was poor reporting on the use and interpretation of MRI before biopsy. MRI is regarded as the imaging method of choice for the detection of spondylodiscitis and the discrimination from other conditions such as non-infectious inflammatory and degenerative disease that may simulate spinal infection [18]. For these reasons, IDSA guidelines recommend performing spine MRI before biopsy in all patients with suspected spondylodiscitis [4–6]. However, 4 studies did not report if MRI was performed [12, 13, 15, 17] and the 4 other studies did not report (clear) MRI criteria for spondylodiscitis [10, 11, 14, 16]. In addition, none of the eight studies included reported the time interval between MRI and biopsy. This rather poor prebiopsy MRI assessment may have negatively affected the culture yields because of potential data contamination with spondylodiscitis mimickers such as Modic type 1 degeneration, acute Schmorl node, and (osteoporotic) fractures [18]. Third, none of the 8 studies reported if patients with a previous history of spondylodiscitis had been excluded. MRI findings in these patients are nonspecific, correlate poorly with clinical and laboratory findings, and may overestimate the diagnosis of spondylodiscitis [19]. This issue may have affected culture yields. Fourth, seven of the eight studies included reported variable anatomical targets for biopsy (disc, vertebral corpus, and/or paravertebral soft tissue) [11–17], whereas one study did not report which anatomical target was biopsied [10]. Variation in anatomical targets may also have affected culture yields. On the other hand, a previous study has shown that there were no statistically significant differences between the yields of endplate-disc, disc-only, and paravertebral soft-tissue biopsies [20]. Despite the variations in patient populations and methodology among the studies included, proportions of positive culture yields among second biopsies were statistically homogeneous, but this may be due to the relatively small sample sizes of the studies included.

This systematic review had several limitations. First, although 93 potentially eligible articles were considered after screening titles and abstracts, only 8 studies with 107 patients who underwent repeat biopsy, remained for analysis. Second, owing to the low number of studies and underreported data, it was not possible to perform further subgroup analyses to determine if any clinical, laboratory, and/or imaging parameters are associated with positive repeat biopsy cultures. Third, although the culture yield of second biopsy was determined, it remains unclear which strategy (i.e., additional blood culture, second biopsy, PEDD, open biopsy, and/or empirical antibiotic therapy without any further diagnostic interventions) is most (cost-)effective in patients with a culture-negative initial biopsy. Thus, future prospective studies with larger sample sizes are needed.

In conclusion, although a second percutaneous image-guided biopsy may have some value in patients with suspected spondylodiscitis, its exact value remains unclear given the poor-quality evidence available. Future well-designed studies are needed to determine the role of a second percutaneous image-guided biopsy in this setting. Such studies should clearly describe the spectrum of patients that was selected for a second percutaneous image-guided biopsy, the method of biopsy, and differences compared with the first biopsy, if any.
